# Evaluation of Assisted Reproductive Technology Health Insurance Coverage for Multiple Pregnancies and Births in Korea

**DOI:** 10.1001/jamanetworkopen.2023.16696

**Published:** 2023-06-06

**Authors:** Wontae Cha, Il Yun, Chung-Mo Nam, Jin Young Nam, Eun-Cheol Park

**Affiliations:** 1 Department of Public Health, Graduate School, Yonsei University, Seoul, Republic of Korea; 2 CHA Hollywood Presbyterian Medical Center, Los Angeles, California; 3 Institute of Health Services Research, Yonsei University, Seoul, Republic of Korea; 4 Department of Preventive Medicine, Yonsei University College of Medicine, Seoul, Republic of Korea; 5 Department of Healthcare Management, Eulji University, Seongnam, Gyeonggi-do, Republic of Korea

## Abstract

**Importance:**

While various policies to support couples experiencing infertility have been introduced due to the fertility rate rapidly dropping in developed countries, few large-scale nationwide cohort studies have evaluated the outcomes of assisted reproductive technology (ART) health insurance coverage policies.

**Objective:**

To evaluate ART health insurance coverage for multiple pregnancies and births in Korea.

**Design, Setting, and Participants:**

This population-based cohort study used delivery cohort data from the Korean National Health Insurance Service database between July 1, 2015, and December 31, 2019. A total of 1 474 484 women were included after exclusion of those who gave birth at nonmedical institutions and those with missing data.

**Exposure:**

Two 27-month periods were examined before and after the Korean National Health Insurance Service had begun covering ART treatment (preintervention period, July 1, 2015, to September 30, 2017; postintervention period, October 1, 2017, to December 31, 2019).

**Main Outcomes and Measures:**

Multiple pregnancies and multiple births were identified by *International Statistical Classification of Diseases and Related Health Problems, Tenth Revision* diagnosis codes. Total births were defined as the total number of babies born to each pregnant woman during the follow-up period. An interrupted time series with segmented regression was conducted to analyze the time trend and its change in outcomes. Data analysis was conducted between December 2, 2022, and February 15, 2023.

**Results:**

Of the 1 474 484 women eligible for the analysis (mean [SD] age, 33.2 [4.6] years), approximately 1.60% had multiple pregnancies and 1.10% had multiple births. After covering ART treatment, the likelihood of multiple pregnancies and multiple births was estimated to increase by 0.7% (estimate, 1.007; 95% CI, 1.004-1.011; *P* < .001) and 1.2% (estimate, 1.012; 95% CI, 1.007-1.016; *P* < .001) compared with before coverage. The probability of an increase in the number of total births per pregnant woman after the intervention was estimated to be 0.5% (estimate, 1.005; 95% CI, 1.005-1.005; *P* < .001). The relatively high-income class above the median income showed a decreasing trend in multiple births and total births before the intervention, but after the intervention, a significant increase was observed.

**Conclusions and Relevance:**

This population-based cohort study found that the possibility of multiple pregnancies and births in Korea significantly increased after the implementation of an ART health insurance coverage policy. These findings suggest that the development and coverage of policies to support couples experiencing infertility may help address low fertility rates.

## Key Points

**Question:**

Was the 2017 health insurance mandate for assisted reproduction associated with increases in multiple pregnancies and births in Korea?

**Findings:**

This cohort study of 1 474 484 women found that the possibility of multiple pregnancies and multiple births increased significantly after coverage for assisted reproductive technology treatment. The number of total births per pregnant woman was on a decreasing trend before the intervention but began to increase after the intervention.

**Meaning:**

These findings suggest that policies that provide financial support for infertility treatment may contribute to addressing Korea’s low birth rates.

## Teaser

This cohort study evaluates the association between health insurance coverage for assisted reproductive technology and pregnancy and birth rates in Korea.

## Introduction

Over the past decades, the average total fertility rate (TFR) has decreased dramatically around the world.^1,2^ The Organization for Economic Co-operation and Development defines TFR as the number of children that a woman would give birth to if she were to live to the end of her childbearing years and give birth to children in alignment with the prevailing fertility rates for each age group.^3^ Assuming no migration and no change in mortality, a TFR of 2.1 children per woman ensures a largely stable population.^3^ As of 2021, the TFR of Organization for Economic Co-operation and Development countries was 1.63.^4^ However, Korea’s TFR is only 0.81, which has resulted in it ranking at the bottom for decades.^5^ The United Nations defines countries with a TFR of 2.1 or less as low-fertility countries and those with a TFR of 1.3 or less as ultra-low-fertility countries.^6^ Accordingly, Korea was already classified as a low-fertility country in 1983 and as an ultra-low-fertility country in 2002.^5^ It is one of the fastest-aging nations in the world.^7^ Since 2020, there has been a reversal in the number of deaths and births, leading to a decline in the population.^5^

South Korea is facing serious problems related to low fertility and an aging population. Various policies are being tried to prevent further population decline and increase the TFR, but the effect has not been significant. To address the complex issues associated with low fertility rates, the Korean government recognized the medical necessity of infertility care and introduced policies of financial support and an assisted reproductive technology (ART) insurance mandate for couples experiencing infertility.^8,9^ The ART health insurance mandate, which has been in effect since October 2017, covers 70% of ART treatment costs to all couples experiencing infertility regardless of income. In the past, people who had incomes lower than the median were given government subsidies for ART treatment, whereas this is the first coverage policy for those with incomes above the median. However, it has not been proven whether government policies supporting ART treatment have contributed to the increase in multiple birth rates and the national TFR.

A previous study in the US found that comprehensive insurance mandates are associated with greater use of ART treatment.^10^ It has also been suggested that health insurance coverage for infertility treatment significantly increases the first birth rate for women older than 35 years.^11^ Several studies have reported on the associations of ART treatment with multiple pregnancies^12,13^ and multiple births.^14,15,16^ Although the cost of ART treatment varies according to each country’s support and coverage policies,^17^ it is still a costly treatment for patients.^18^ Since Korea began to provide coverage for ART in 2017, few studies have evaluated the association of covering ART with patients’ economic burden, health outcomes, and fertility rates.

Therefore, this study aimed to evaluate whether the ART insurance mandate is associated with the increase in multiple pregnancies and births in Korea. Our hypothesis was that the probability of having multiple pregnancies and births would rise, as well as the total number of live births per pregnant woman, after the intervention.

## Methods

### Data and Study Population

This population-based cohort study used the Korean National Health Insurance Service (NHIS) database. In 1989, universal health coverage was introduced in Korea, which made it compulsory for all citizens to subscribe to the NHIS. Consequently, health insurance covers approximately 98% of the total population. The NHIS database contains all Koreans’ health screening data, medical utilization claims data, sociodemographic characteristics, and death information.^19,20^ The claims data comprise the largest database provided by the NHIS, which contains data on all medical use history for the entire Korean population, including diagnosis codes according to the clinically determined *International Statistical Classification of Diseases and Related Health Problems, Tenth Revision* (*ICD-10*), drug prescriptions, length of hospital stay, medical expenses, and information on health care provision.^20^

The NHIS delivery cohort database we obtained included information on all women who have experienced childbirth at least once at Korean medical institutions between July 1, 2015, and December 31, 2019. Delivery was defined using any record of inpatient hospitalization that included pregnancy-related diagnosis codes or procedure codes for vaginal or cesarean deliveries.^21^ A total of 1 474 484 individuals constituted the study population after excluding those with no birth records at Korean medical institutions and those with missing data.

The study protocol was approved by the institutional review board of Eulji University (No. EU22-27). Because the NHIS database we used did not contain identifiable information, the requirement for informed consent was waived. In conducting this observational study, we followed the Strengthening the Reporting of Observational Studies in Epidemiology (STROBE) reporting guideline for cohort studies.

### Variables

The main variable of interest was the time of introduction of the ART health insurance coverage policy, which was divided into the periods before and after the intervention. Because the NHIS began covering ART on October 1, 2017, and the last follow-up date for data was December 31, 2019, we determined the follow-up periods before and after the intervention to be 27 months each. Therefore, the preintervention period was from July 1, 2015, to September 30, 2017 and the postintervention period was from October 1, 2017, to December 31, 2019. Study participants were classified as preintervention or postintervention based on the time of their delivery. Whether or not participants received ART could only be identified during the postintervention period because ART has only been recorded in claims data since October 2017, when the ART insurance mandates were implemented. Therefore, after the intervention, ART could be defined as infertility treatment (*ICD-10* code N97) using all ART procedures, including intrauterine insemination (IUI) (*ICD-10* code Z31.1) and in vitro fertilization and embryo transfer (IVF-ET) (*ICD-10* code Z31.2).^21,22^

There were 3 dependent variables. First, multiple pregnancies were cases in which 2 or more fetuses were conceived and were identified with *ICD-10* code O30. Second, multiple births were cases in which 2 or more babies were born and were determined by *ICD-10* code O84. Third, total births were defined as the total number of babies born to each pregnant woman during the follow-up period and were classified into categories from 1 to 5.

As covariates, we included sociodemographic and obstetric factors. Sociodemographic factors were maternal age (<20, 20-29, 30-39, and ≥40 years), region (Seoul, metropolitan areas, small cities, and rural areas), income level (quartile 1 [lowest] to quartile 4 [highest]), and type of insurance (regionally insured, workplace insured, and medical aid). Obstetric factors were parity (nulliparous and multiparous) and maternal comorbidities (0 or ≥1).^23^

### Statistical Analysis

Statistical analysis was conducted between December 2, 2022, and February 15, 2023. Descriptive statistics are presented as frequencies and percentages. To investigate and compare the general characteristics of the study population, a χ^2^ test was conducted. Subsequently, an interrupted time series (ITS) with segmented regression was performed to analyze the time trend and its change in outcomes. The ITS is modeled using a linear regression model, including 3 time-related variables, and the regression coefficients estimate the preintervention slope, level change at the time of the intervention, and postintervention slope change. The slope change quantifies the difference between the preintervention and postintervention slopes. Level change represents an absolute change in the level of outcomes at the time of the intervention, which measures the immediate effect of the intervention.^24^ Because we applied the log-link function to the generalized linear model to perform segmented regression, the model coefficients had to be converted into exponentials to represent trends and changes in the outcomes on the original scale. To interpret the model coefficients, log[E(*Y*_i_)] needed to be converted into multiplicative interpretations for the original scale E(*Y*_i_) = *μ_i_*:

log(*μ_i_*) = *β_0_* + *β_1_* × time_t_ + *β_2_* × intervention_t_ + *β_3_* × time after intervention_t_ + *e_t_*

In this model, the intercept *β_0_* estimates the baseline level of the outcome; *β_1_* estimates the baseline trend of the outcome; *β_2_* estimates the level change after the intervention, which indicates the immediate effect size of the intervention; *β_3_* estimates the change in trend after the intervention; and the sum of *β_1_* and *β_3_* is the slope after the intervention, indicating the follow-up outcome trend.^25^ Parameter estimates, SEs, and *P* values are presented as key results.

Subsequently, a modified Poisson regression analysis with sandwich error estimation was performed to examine the association between ART treatment and outcomes after the intervention using adjusted risk ratios and 95% CIs. SAS, version 9.4 statistical software (SAS Institute Inc) was used for all analyses. A 2-sided *P* < .05 was considered statistically significant.

## Results

Table 1 presents the general characteristics of the participants. Of the 1 474 484 women eligible for the analysis (mean [SD] age, 33.2 [4.6] years), those in their 30s (1 077 501 [73.1%]), living in small cities (732 297 [49.7%]), and workplace insured (1 168 841 [79.3%]) were the most reported. Among the study population, those who experienced multiple pregnancies and multiple births were 1.60% and 1.10%, respectively. During the entire follow-up period, 73.54% of the women gave birth to only 1 child, and it was extremely rare for any woman to give birth to more than 3 children (0.02%). The total number of mothers after the intervention (n = 653 386) decreased compared with before the intervention (n = 821 098). However, the proportion of multiple pregnancies and multiple births increased after the intervention to 1.75% and 1.15%, respectively. Similarly, the proportion of women having 2 or more children increased during the postintervention period (28.44% vs 24.87% preintervention). eTable 1 in Supplement 1 shows the demographic characteristics of the study population by preintervention and postintervention periods.

**Table 1.** General Characteristics of the Study Population

Variable	Total, No. (N = 1 474 484)	Multiple pregnancies			Multiple births			Total births per pregnant woman					
		No. (%)		*P* value	No. (%)		*P* value	No. (%)					*P* value
		Yes	No		Yes	No		1	2	3	4	5	
ART health insurance coverage													
Before intervention (7/1/2015 to 9/30/2017)	821 098	12 090 (1.47)	809 008 (98.53)	<.001	8651 (1.05)	812 447 (98.95)	<.001	616 884 (75.13)	196 805 (23.97)	7261 (0.88)	146 (0.02)	2 (<0.01)	<.001
After intervention (10/1/2017 to 12/31/2019)	653 386	11 464 (1.75)	641 922 (98.25)		7503 (1.15)	645 883 (98.85)		467 480 (71.55)	178 863 (27.37)	6887 (1.05)	153 (0.02)	3 (<0.01)	
Maternal age, y													
<20	3941	21 (0.53)	3920 (99.47)	<.001	14 (0.36)	3927 (99.64)	<.001	3120 (79.17)	760 (19.28)	59 (1.50)	2 (0.05)	0	<.001
20-29	328 306	2752 (0.84)	325 554 (99.16)		1902 (0.58)	326 404 (99.42)		223 433 (68.06)	100 288 (30.55)	4461 (1.36)	124 (0.04)	0	
30-39	1 077 501	19 518 (1.81)	1 057 983 (98.19)		13 456 (1.25)	1 064 045 (98.75)		801 689 (74.40)	266 307 (24.72)	9336 (0.87)	167 (0.02)	2 (<0.01)	
≥40	64 736	1263 (1.95)	63 473 (98.05)		782 (1.21)	63 954 (98.79)		56 122 (86.69)	8313 (12.84)	292 (0.45)	6 (0.01)	3 (<0.01)	
Region													
Seoul	281 795	5552 (1.97)	276 243 (98.03)	<.001	3393 (1.20)	278 402 (98.80)	<.001	215 066 (76.32)	64 691 (22.96)	1989 (0.71)	49 (0.02)	0	<.001
Metropolitan areas	383 187	5707 (1.49)	377 480 (98.51)		4848 (1.27)	378 339 (98.73)		280 299 (73.15)	99 006 (25.84)	3819 (1.00)	63 (0.02)	0	
Small cities	732 297	11 250 (1.54)	721 047 (98.46)		7254 (0.99)	725 043 (99.01)		535 095 (73.07)	189 908 (25.93)	7132 (0.97)	162 (0.02)	0	
Rural	77 205	1045 (1.35)	76 160 (98.65)		659 (0.85)	76 546 (99.15)		53 904 (69.82)	22 063 (28.58)	1208 (1.56)	25 (0.03)	5 (0.01)	
Income quartile^a^													
1 (Lowest)	299 343	4317 (1.44)	295 026 (98.56)	<.001	2986 (1.00)	296 357 (99.00)	<.001	211 219 (70.56)	84 189 (28.12)	3839 (1.28)	94 (0.03)	2 (<0.01)	.001
2	337 489	4261 (1.26)	333 228 (98.74)		3036 (0.90)	334 453 (99.10)		247 764 (73.41)	86 209 (25.54)	3429 (1.02)	84 (0.02)	3 (<0.01)	
3	524 584	8494 (1.62)	516 090 (98.38)		5880 (1.12)	518 704 (98.88)		388 963 (74.15)	131 136 (25.00)	4425 (0.84)	60 (0.01)	0	
4 (Highest)	313 068	6482 (2.07)	306 586 (97.93)		4252 (1.36)	308 816 (98.64)		236 418 (75.52)	74 134 (23.68)	2455 (0.78)	61 (0.02)	0	
Type of insurance													
Regional	296 390	3899 (1.32)	292 491 (98.68)	<.001	2685 (0.91)	293 705 (99.09)	<.001	220 993 (74.56)	71 760 (24.21)	3560 (1.20)	76 (0.03)	1 (<0.01)	<.001
Workplace	1 168 841	19 574 (1.67)	1 149 267 (98.33)		13 420 (1.15)	1 155 421 (98.85)		856 542 (73.28)	301 723 (25.81)	10 374 (0.89)	198 (0.02)	4 (<0.01)	
Medical aid	9253	81 (0.88)	9172 (99.12)		49 (0.53)	9204 (99.47)		6829 (73.80)	2185 (23.61)	214 (2.31)	25 (0.27)	0	
Parity													
Nulliparous	785 047	17 727 (2.26)	767 320 (97.74)	<.001	11 871 (1.51)	773 176 (98.49)	<.001	613 744 (78.18)	166 484 (21.21)	4722 (0.60)	97 (0.01)	0	<.001
Multiparous	689 437	5827 (0.85)	683 610 (99.15)		4283 (0.62)	685 154 (99.38)		470 620 (68.26)	209 184 (30.34)	9426 (1.37)	202 (0.03)	5 (<0.01)	
Maternal comorbidities													
0	571 903	7595 (1.33)	564 308 (98.67)	<.001	5263 (0.92)	566 640 (99.08)	<.001	414 823 (72.53)	151 241 (26.45)	5703 (1.00)	135 (0.02)	1 (<0.01)	<.001
≥1	902 581	15 959 (1.77)	886 622 (98.23)		10 891 (1.21)	891 690 (98.79)		669 541 (74.18)	224 427 (24.87)	8445 (0.94)	164 (0.02)	4 (<0.01)	
Delivery year													
2015	193 697	2794 (1.44)	190 903 (98.56)	<.001	2038 (1.05)	191 659 (98.95)	<.001	138 335 (71.42)	52 955 (27.34)	2368 (1.22)	38 (0.02)	1 (<0.01)	<.001
2016	372 965	5539 (1.49)	367 426 (98.51)		4020 (1.08)	368 945 (98.92)		284 924 (76.39)	85 410 (22.90)	2574 (0.69)	56 (0.02)	1 (<0.01)	
2017	328 614	4979 (1.52)	323 635 (98.48)		3400 (1.03)	325 214 (98.97)		248 433 (75.60)	77 071 (23.45)	3041 (0.93)	69 (0.02)	0	
2018	300 768	5001 (1.66)	295 767 (98.34)		3207 (1.07)	297 561 (98.93)		219 965 (73.13)	78 277 (26.03)	2461 (0.82)	63 (0.02)	2 (<0.01)	
2019	278 440	5241 (1.88)	273 199 (98.12)		3489 (1.25)	274 951 (98.75)		192 707 (69.21)	81 955 (29.43)	3704 (1.33)	73 (0.03)	1 (<0.01)	
Total	1 474 484	23 554 (1.60)	1 450 930 (98.40)		16 154 (1.10)	1 458 330 (98.90)		1 084 364 (73.54)	375 668 (25.48)	14 148 (0.96)	299 (0.02)	5 (<0.01)	

Abbreviation: ART, assisted reproductive technology.

^a^ Income level was classified into quartiles according to monthly household gross income (quartile 1, <$1950; quartile 2, $1950-$3899; quartile 3, $3900-$5849; quartile 4, ≥$5850).

Table 2 presents the results of the segmented regression analysis used to estimate the probabilities of the 3 outcome variables, adjusting for all covariates. The likelihood of having multiple pregnancies after ART insurance coverage was estimated to increase by 0.7% compared with before coverage (estimate, 1.007; 95% CI, 1.004-1.011; *P* < .001). Multiple births during the preintervention period showed a decreasing trend (estimate, 0.997; 95% CI, 0.994-0.999; *P* = .02) but changed to an increasing trend after the intervention (estimate, 1.008; 95% CI, 1.005-1.011; *P* < .001). The probability of having multiple births after the intervention increased by 1.2% compared with before the intervention (estimate, 1.012; 95% CI, 1.007-1.016; *P* < .001). In addition, the total number of births per pregnant woman increased by 0.5% (estimate, 1.005; 95% CI, 1.005-1.005; *P* < .001) with ART coverage. Before the intervention, there was a downward trend in the number of births per woman (estimate, 0.998; 95% CI, 0.998-0.998; *P* < .001), but after the intervention, there was a significant increase (estimate, 1.003; 95% CI, 1.003-1.003; *P* < .001).

**Table 2.** Segmented Regression Models Estimating the Probability of Multiple Pregnancies, Multiple Births, and Total Births Per Pregnant Woman

	Estimate^a^	SE	95% CI	*P* value
**Multiple pregnancies**				
Intercept *β_0_*	0.004	0.036	0.004-0.005	<.001
Baseline outcome trend *β_1_*	1.000	0.001	0.998-1.003	.81
Level change after intervention *β_2_*	1.037	0.027	0.983-1.093	.19
Trend change after intervention *β_3_*	1.007	0.002	1.004-1.011	<.001
Follow-up outcome trend *β_1_* + *β_3_*	1.008	0.001	1.005-1.010	<.001
**Multiple births**				
Intercept *β_0_*	0.003	0.043	0.003-0.003	<.001
Baseline outcome trend *β_1_*	0.997	0.001	0.994-0.999	.02
Level change after intervention *β_2_*	0.987	0.033	0.925-1.053	.70
Trend change after intervention *β_3_*	1.012	0.002	1.007-1.016	<.001
Follow-up outcome trend *β_1_* + *β_3_*	1.008	0.002	1.005-1.011	<.001
**Total births per pregnant woman**				
Intercept *β_0_*	4.074	1.002	4.060-4.089	<.001
Baseline outcome trend *β_1_*	0.998	1.000	0.998-0.998	<.001
Level change after intervention *β_2_*	1.026	1.002	1.023-1.029	<.001
Trend change after intervention *β_3_*	1.005	1.000	1.005-1.005	<.001
Follow-up outcome trend *β_1_* + *β_3_*	1.003	1.000	1.003-1.003	<.001

^a^ Estimates were calculated by statistically adjusting for all covariates. Definitions of *β_0_*, *β_1_*, *β_2_*, and *β_3_* appear in the Statistical Analysis section.

As shown in Table 2, the size of the level and trend changes could be quantitatively confirmed by calculating the parameter estimates, whereas as seen in the Figure, it was possible to intuitively confirm the outcome trend after the intervention. The ITS results showed that multiple pregnancies, multiple births, and total births increased after ART health insurance coverage was introduced.

**Figure.**
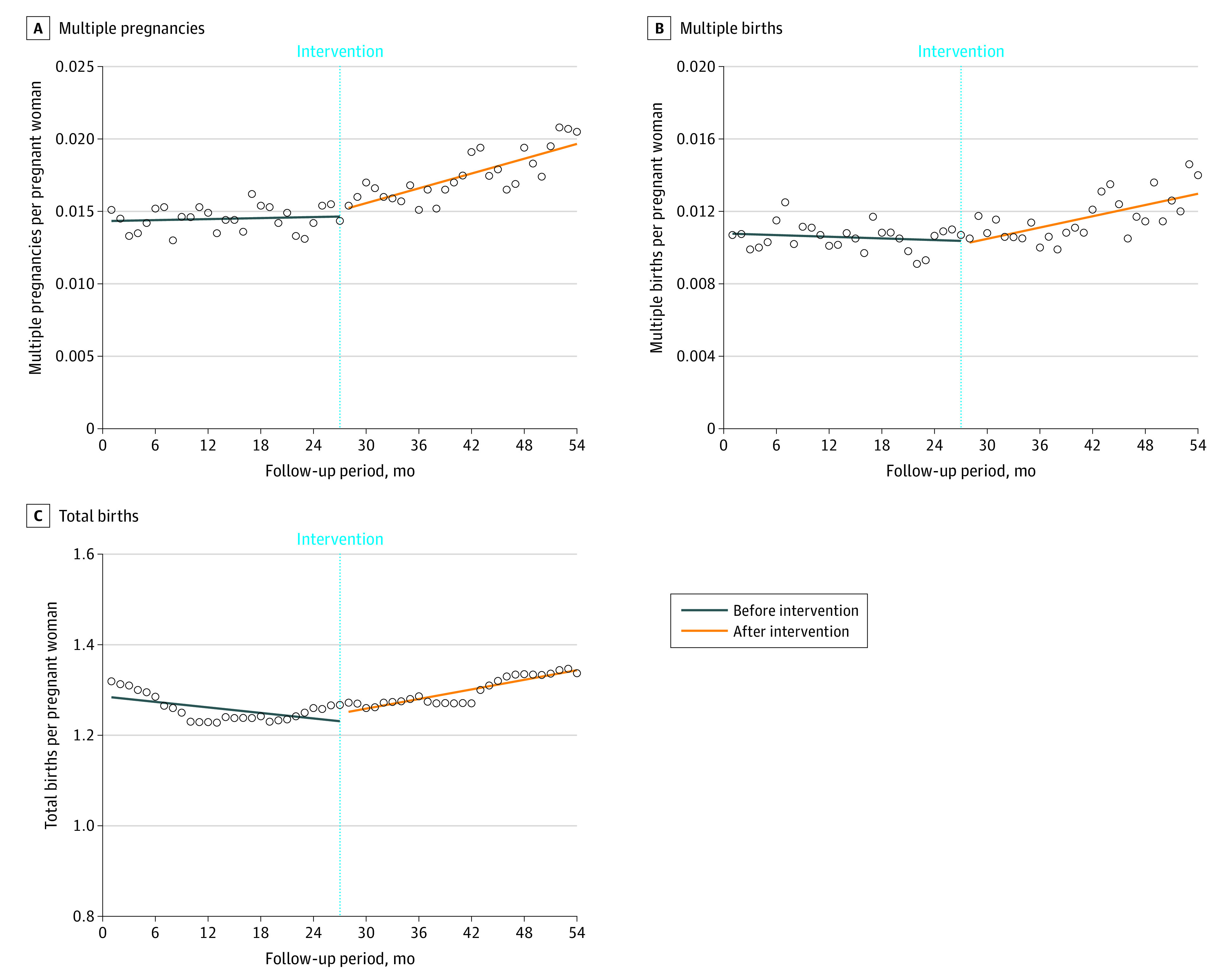
Factors Associated With Trends of Multiple Pregnancies, Multiple Births, and Total Births Per Pregnant Woman Circles represent observed values.

As Table 3 shows, subgroup analyses stratified by income level were conducted considering that the benefits of ART treatment subsidies differed according to income level. Similarly, all remaining covariates other than income level were statistically adjusted to calculate the estimates. Notably, the groups with income levels in quartiles 3 and 4 showed a decreasing trend in multiple births and total births before ART coverage that increased significantly after the intervention (quartile 3: trend change after intervention, 1.013; 95% CI, 1.007-1.020 [*P* < .001] and 1.005; 95% CI, 1.005-1.005 [*P* < .001], respectively; quartile 4: trend change after intervention, 1.011; 95% CI, 1.003-1.019 [*P* = .009] and 1.006; 95% CI, 1.005-1.006 [*P* < .001], respectively).

**Table 3.** Results of Subgroup Analysis Stratified by Income Level^a^

	Estimate (95% CI)^b^							
	Quartile 1 (Lowest)	*P* value	Quartile 2	*P* value	Quartile 3	*P* value	Quartile 4 (Highest)	*P* value
**Multiple pregnancies**								
Intercept	0.003 (0.003-0.004)	<.001	0.003 (0.003-0.003)	<.001	0.003 (0.003-0.003)	<.001	0.004 (0.003-0.004)	<.001
Baseline outcome trend	1.003 (0.998-1.008)	.30	1.000 (0.994-1.005)	.88	1.001 (0.997-1.005)	.74	0.998 (0.994-1.003)	.39
Level change after intervention	0.990 (0.877-1.119)	.88	1.012 (0.892-1.147)	.86	1.009 (0.923-1.104)	.84	1.115 (1.006-1.236)	.04
Trend change after intervention	1.003 (0.996-1.011)	.39	1.011 (1.003-1.019)	.008	1.010 (1.004-1.016)	.001	1.006 (0.999-1.012)	.09
Follow-up outcome trend	1.006 (1.001-1.012)	.03	1.010 (1.005-1.016)	<.001	1.010 (1.006-1.015)	<.001	1.004 (0.999-1.009)	.13
**Multiple births**								
Intercept	0.003 (0.002-0.003)	<.001	0.002 (0.002-0.003)	<.001	0.002 (0.002-0.003)	<.001	0.007 (0.006-0.008)	<.001
Baseline outcome trend	0.997 (0.991-1.003)	.34	1.002 (0.996-1.008)	.57	0.995 (0.990-1.000)	.03	0.996 (0.990-1.001)	.10
Level change after intervention	0.936 (0.806-1.086)	.38	0.929 (0.801-1.078)	.33	0.996 (0.894-1.110)	.95	1.053 (0.929-1.195)	.42
Trend change after intervention	1.013 (1.004-1.023)	.005	1.008 (0.999-1.018)	.08	1.013 (1.007-1.020)	<.001	1.011 (1.003-1.019)	.009
Follow-up outcome trend	1.010 (1.004-1.017)	.003	1.010 (1.004-1.017)	.003	1.008 (1.003-1.013)	<.001	1.006 (1.000-1.012)	.04
**Total births per pregnant woman**								
Intercept	4.261 (4.227-4.295)	<.001	4.399 (4.366-4.432)	<.001	4.076 (4.052-4.110)	<.001	3.898 (3.868-3.929)	<.001
Baseline outcome trend	1.000 (0.999-1.000)	.003	0.996 (0.996-0.997)	<.001	0.998 (0.998-0.998)	<.001	1.000 (0.999-1.000)	<.001
Level change after intervention	1.036 (1.029-1.042)	<.001	1.009 (1.003-1.015)	<.001	1.025 (1.021-1.030)	<.001	1.032 (1.025-1.038)	<.001
Trend change after intervention	1.004 (1.003-1.004)	<.001	1.005 (1.004-1.005)	<.001	1.005 (1.005-1.005)	<.001	1.006 (1.005-1.006)	<.001
Follow-up outcome trend	1.003 (1.003-1.003)	<.001	1.001 (1.001-1.001)	<.001	1.003 (1.003-1.003)	<.001	1.005 (1.005-1.005)	<.001

^a^ Income level was classified into quartiles according to monthly household gross income (quartile 1, <$1950; quartile 2, $1950-$3899; quartile 3, $3900-$5849; quartile 4, ≥$5850).

^b^ Estimates were calculated by statistically adjusting for all remaining covariates other than income level.

We also performed a Poisson regression analysis to examine the association between ART treatment and outcomes after the intervention (eTable 2 in Supplement 1). Compared with mothers not treated with ART, adjusted risk ratios show that those who received IUI and IVF-ET treatment were 13.67 times (95% CI, 13.11-14.26) and 11.27 times (95% CI, 10.50-12.10) more likely to have multiple pregnancies, respectively. Similarly, women who received IUI and IVF-ET treatment were 13.64 times (95% CI, 12.94-14.39) and 11.46 times (95% CI, 10.47-12.53) more likely to give birth to multiple babies, respectively. The number of total births per mother was 1.07 times (95% CI, 1.07-1.08) higher for mothers who received IUI treatment and 1.04 times (95% CI, 1.03-1.05) higher for those who received IVF-ET treatment than for mothers who did not receive any ART treatment.

## Discussion

This cohort study evaluated the demographic policy outcomes of ART treatment coverage introduced as a solution to Korea’s low fertility rate, which is the lowest in the world.^5^ There were 3 principal findings. First, since October 2017, 70% of the total expenses of ART treatment were covered by health insurance for all couples experiencing infertility regardless of income level, and the possibility of multiple pregnancies and multiple births increased significantly. Second, the number of total births per pregnant woman showed a decreasing trend before the intervention but increased after the intervention. Before ART coverage, planning additional births through ART procedures would be challenging due to the high cost of infertility treatment. However, it is anticipated that after coverage, the cost burden has lessened, leading to a relative increase in the total number of births. Third, in high-income groups that were not eligible for financial support for ART treatment before the intervention, the introduction of ART insurance mandates had a more noticeable association with multiple births and total births. We believe that health insurance coverage for ART positively contributed to multiple pregnancies, multiple births, and total births. Although it was not a complete coverage and the out-of-pocket expense was 30% of the total treatment cost, the outcomes of introducing the policy were remarkable. According to NHIS claims data, the proportion of newborns conceived through ART procedures in Korea has actually increased over the past 5 years since the implementation of ART health insurance coverage, reaching 12.3% in 2022.^26^ As the age of marriage and first childbirth is delayed, the proportion of couples experiencing infertility and the use of ART treatment are expected to increase.

Most previous studies that examined the outcomes of ART insurance mandates focused on economic burden,^17,18^ use of ART treatment,^27^ and pregnancy outcomes.^11,28^ According to their findings, implementing ART coverage policies reduces patient costs and is positively associated with ART treatment use and pregnancy outcomes. There was also evidence that ART contributes to improving fertility rates and demographic changes.^29^ The findings of these studies are consistent with the implications of our study: ART should be actively supported as a national strategy to address low fertility rates. On the other hand, several studies have reported adverse associations of ART with maternal health outcomes. For example, a higher risk of severe maternal morbidity has been confirmed in mothers treated with ART than in those not treated with ART.^21^

The biggest difference between previous research and our study is that we not only evaluated the outcomes of ART treatment but also the outcomes of the ART treatment coverage policy. Considering that ART treatment imposes a very large economic burden on couples experiencing infertility,^18^ it is important to cover it under the NHIS. Korea’s 2017 ART insurance mandates targeted all couples experiencing infertility regardless of income. People with incomes below the median had previously received government subsidies for ART treatment, but the 2017 coverage policy was the first to cover people with incomes above the median. Therefore, in this study, differences in intervention outcomes according to income level were observed. This policy also contributed to an increase in total births per woman, which had previously been on a downward trend. Although Korea’s TFR continues to decline, our findings suggest that the decline in TFR may be slowed slightly by the ART health insurance coverage policy.

### Strengths and Limitations

This study has several strengths. A major strength is that the NHIS database we analyzed contains nationwide cohort data, which ensures its applicability to the study when assessing the outcomes of medical practice and health. In addition, we used the ITS design, which is a strong quasi-experimental approach for evaluating the longitudinal outcomes of interventions.^30^ The main advantage of this design is that it makes full use of the longitudinal nature of the data and accounts for preintervention trends.^24^ Previous studies have used a difference-in-differences study design that compares only 2 time points to investigate the net policy association with the outcomes^31^ or segmented regression with fewer than 10 time points.^32^ However, there were too few time points to capture baseline trends and changes. This study used 54 time points (27 time points each before and after the intervention) to capture trend changes more robustly.

This study also had several limitations. First, the NHIS delivery cohort data we analyzed included only information on women who had experienced childbirth during the study period and did not include information on all women of childbearing potential in Korea; therefore, it was not possible to explore the outcomes of changes in the TFR trend with all Korean women of childbearing age as the denominator. Second, the inherent limitations of administrative claims data have been pointed out. Korea’s NHIS cohort data set is not a medical record; rather, it provides data for requesting the insurer to pay a part of patients’ total medical expenses.^20^ Thus, the recorded disease codes in the NHIS may not represent the actual disease status of patients.^20^ Moreover, it was not possible to verify whether ART treatment received an uninsured benefit. Third, we used the *ICD-10* codes to select study participants and identify their specific outcomes, but these codes inherently have some problems.^33,34,35^ The *ICD-10* codes are primarily used for administrative purposes and may not provide detailed clinical information about the patient. There is also the concern of incomplete coding, which could misclassify or underestimate the outcomes. Fourth, we could not control for all of the other informal benefits and interventions provided to couples experiencing infertility around the same time as the ART health insurance coverage policy was introduced. Thus, although there may have been an overestimation of the associations, ART insurance coverage was the most comprehensive and official intervention promoted at that time. Fifth, the follow-up period is insufficient for assessing the actual outcomes of the policy because certain patients may have undergone IVF before the intervention and transferred embryos at different times, leading to variability in the follow-up period, which may not truly reflect the policy’s impact. Sixth, we tried to adjust for potential confounders associated with childbirth, such as parity and maternal comorbidities, but residual confounding from unmeasured variables could not be ruled out.

## Conclusions

The findings of this population-based cohort study suggest that ART health insurance coverage is associated with an increase in multiple pregnancies and multiple births and may also contribute to improving the birth rate. Considering the demographic structure of Korea, which has a very low fertility rate, policies that can support couples experiencing infertility should be further developed and implemented.

## Notes

Supplement 1. **eTable 1.** Demographic Characteristics of the Study Population Before and After Intervention **eTable 2.** Association Between ART Treatment and Outcomes (After Intervention)

Supplement 2.
Data Sharing Statement
