# Supplementary material for: Evaluation of Assisted Reproductive Technology Health Insurance Coverage for Multiple Pregnancies and Births in Korea
Source: JAMA Netw Open. 2023 Jun 6;6(6):e2316696. doi: 10.1001/jamanetworkopen.2023.16696 (PMC10245192; doi:10.1001/jamanetworkopen.2023.16696)
Supplement: Supplement 1. — eTable 1. Demographic Characteristics of the Study Population Before and After Intervention eTable 2. Association Between ART Treatment and Outcomes (After Intervention) [file jamanetwopen-e2316696-s001.pdf]

Supplemental Online Content

Cha W, Yun I, Nam CM, Nam JY, Park EC. Evaluation of assisted reproductive technology health insurance coverage for multiple pregnancies and births in Korea. *JAMA Netw Open*. 2023;6(6):e2316696. doi:10.1001/jamanetworkopen.2023.16696

eTable 1. Demographic Characteristics of the Study Population Before and After Intervention

eTable 2. Association Between ART Treatment and Outcomes (After Intervention)

This supplemental material has been provided by the authors to give readers additional information about their work.

© 2023 Cha W et al. *JAMA Network Open*.

eTable 1. Demographic Characteristics of the Study Population Before and After Intervention

Variables	Total	Before intervention (2015.07 ~ 2017.09)		After intervention (2017.10 ~ 2019.12)	
	n	n	%	n	%
	1,474,484	821,098	100.00	653,386	100.00
**Maternal age (years)**					
< 20	3,941	2,374	0.29	1,567	0.24
20-29	328,306	188,124	22.91	140,182	21.45
30-39	1,077,501	595,030	72.47	482,471	73.84
≥ 40	64,736	35,570	4.33	29,166	4.46
**Region**					
Seoul	281,795	156,165	19.02	125,630	19.23
Metropolitans	383,187	211,586	25.77	171,601	26.26
Small cities	732,297	409,854	49.92	322,443	49.35
Rural	77,205	43,493	5.30	33,712	5.16
**Income level**[a]					
Quartile 1	299,343	168,946	20.58	130,397	19.96
Quartile 2	337,489	190,198	23.16	145,041	22.20
Quartile 3	524,584	289,106	35.21	238,478	36.50
Quartile 4	313,068	172,848	21.05	141,720	21.69
**Type of insurance**					
Regionally-Insured	296,390	167,051	20.34	129,339	19.80
Workplace-Insured	1,168,841	648,404	78.97	520,437	79.65
Medical aids	9,253	5,643	0.69	3,610	0.55
**Parity**					
Nulliparous	785,047	436,170	53.12	348,877	53.40
Multiparous	689,437	384,928	46.88	304,509	46.60
**Maternal comorbidities**					
0	571,903	318,976	38.85	252,927	38.71
1 +	902,581	502,122	61.15	400,459	61.29

[a] Income level was classified into quartiles according to monthly household gross income (Quartile 1: less than about $1,950, Quartile 2: about $1,950~$3,900, Quartile 3: about $3,900~$5,850, Quartile 4: about $5,850 and up)

© 2023 Cha W et al. *JAMA Network Open*.

eTable 2. Association Between ART Treatment and Outcomes (After Intervention)

	Multiple Pregnancies		Multiple Births		Total Births	
	Adjusted RR[a]	95% CI	Adjusted RR[a]	95% CI	Adjusted RR[a]	95% CI
**ART**						
Non-ART[b]	1.00		1.00		1.00	
IUI	13.67	(13.11 - 14.26)	13.64	(12.94 - 14.39)	1.07	(1.07 - 1.08)
IVF-ET	11.27	(10.50 - 12.10)	11.46	(10.47 - 12.53)	1.04	(1.03 - 1.05)
**Maternal age (years)**						
< 20	0.69	(0.36 - 1.32)	0.47	(0.17 - 1.26)	0.98	(0.97 - 1.00)
20-29	1.00		1.00		1.00	
30-39	1.53	(1.43 - 1.62)	1.52	(1.41 - 1.64)	0.95	(0.94 - 0.95)
≥ 40	1.26	(1.15 - 1.38)	1.15	(1.02 - 1.29)	0.83	(0.82 - 0.83)
**Region**						
Seoul	1.00		1.00		1.00	
Metropolitans	0.83	(0.79 - 0.88)	1.19	(1.12 - 1.27)	1.00	(1.00 - 1.01)
Small cities	0.93	(0.89 - 0.97)	1.00	(0.94 - 1.06)	1.00	(1.00 - 1.00)
Rural	0.96	(0.88 - 1.05)	1.12	(1.00 - 1.26)	1.01	(1.01 - 1.02)
**Income level[c]**						
Quartile 1	0.90	(0.85 - 0.95)	0.93	(0.87 - 0.99)	1.02	(1.02 - 1.03)
Quartile 2	0.83	(0.79 - 0.88)	0.89	(0.83 - 0.96)	1.01	(1.00 - 1.01)
Quartile 3	0.92	(0.88 - 0.97)	0.94	(0.88 - 0.99)	1.01	(1.01 - 1.01)
Quartile 4	1.00		1.00		1.00	
**Type of insurance**						
Regionally-Insured	0.93	(0.89 - 0.98)	0.98	(0.92 - 1.04)	0.98	(0.98 - 0.98)
Workplace-Insured	1.00		1.00		1.00	
Medical aids	1.05	(0.77 - 1.42)	0.92	(0.62 - 1.36)	0.92	(0.91 - 0.94)
**Parity**						
Nulliparous	1.85	(1.77 - 1.94)	1.73	(1.63 - 1.83)	0.67	(0.67 - 0.67)
Multiparous	1.00		1.00		1.00	
**Obstetric comorbidities**						
0	1.00		1.00		1.00	
1 +	1.10	(1.05 - 1.14)	1.13	(1.08 - 1.19)	0.99	(0.99 - 1.00)

[a] Adjusted RRs (Risk ratio) were adjusted for all covariates

[b] Non-ART set as the reference group included those who gave birth trough natural conception without undergoing any ART treatment

[C] Income level was classified into quartiles according to monthly household gross income (Quartile 1: less than about $1,950, Quartile 2: about $1,950~$3,900, Quartile 3: about $3,900~$5,850, Quartile 4: about $5,850 and up)

© 2023 Cha W et al. *JAMA Network Open*.
