# Supplementary material for: Evaluation of Assisted Reproductive Technology Health Insurance Coverage for Multiple Pregnancies and Births in Korea
Source: JAMA Netw Open. 2023 Jun 6;6(6):e2316696. doi: 10.1001/jamanetworkopen.2023.16696 (PMC10245192; doi:10.1001/jamanetworkopen.2023.16696)
Supplement: Supplement 2. — Data Sharing Statement [file jamanetwopen-e2316696-s002.pdf]

Data Sharing Statement

Cha. Evaluation of Assisted Reproductive Technology Health Insurance Coverage for Multiple Pregnancies and Births in Korea. *JAMA Netw Open*. Published June 06, 2023. doi:10.1001/jamanetworkopen.2023.16696

Data
Data available: No
